# Nanotechnology in dentistry: Bridging science and practice

**DOI:** 10.6026/973206300210522

**Published:** 2025-03-31

**Authors:** Anamika Shrikrishna Deshpande, Akash Daddanala, Maninderjit Kaur, Nagham Awad Chahen, Madhuriben Patel, Nikitha Tummeti

**Affiliations:** 1 Department of Dental Surgery, Dr. D.Y. Patil Dental college and Hospital, Mumbai, Maharashtra, India; 2 Department of Dental Surgery, Sri Sai College Of Dental Surgery, Telangana, India; 3 Department of Dental Surgery, Adesh Institute of Dental Sciences and Research, Punjab, India; 4 Department of Dental Surgery, Hama University, Hama, Syria; 5 Department of Dental Surgery, Narsinhbhai Patel Dental College and Hospital, Gujarat, India

**Keywords:** Nanotechnology, dentistry, dental diagnosis, restorative dentistry, endodontics, implantology, periodontics, drug delivery, tissue engineering, nano-composites, dental implants, dental regeneration, dental biomaterials, osseointegration

## Abstract

Nanotechnology enhances diagnostic precision in dentistry using nano-sensors and advanced imaging agents. Nano-composites improve the mechanical properties of restorative materials by enhancing durability and aesthetics. Nanoparticles facilitate efficient root canal disinfection by increasing antimicrobial efficacy in endodontics application. Nano-modified implant surfaces promote better Osseo-integration and reduce infection risk in implantology. Moreover, nanotechnology-driven tissue engineering enables dental regeneration through nano-engineered scaffolds that support cell growth and differentiation.

## Background:

The integration of nanotechnology in dentistry represents a revolutionary advancement in dental materials, diagnosis and treatment methodologies and offering significant improvements in precision, efficacy and biocompatibility [1]. Nanotechnology, defined as the manipulation of matter at the atomic or molecular scale, enables the creation of innovative materials and devices with enhanced properties that were previously unattainable [2]. The role of nanotechnology in diagnostic tools has opened new avenues for early detection of dental diseases, which is crucial for successful treatment [3]. Nanotechnology-based sensors can detect early signs of tooth decay, periodontal disease and oral cancers at a molecular level, often before they are visible to the naked eye or detectable using conventional imaging techniques. Nanoparticles can be functionalized to bind specifically to disease biomarkers, allowing for non-invasive, accurate and rapid diagnostics [4]. Nanomaterials, due to their unique size and surface properties, have transformed the development of dental materials [5]. In restorative dentistry, nano-composites are now used for fillings, offering superior strength, wear resistance and aesthetic qualities when compared to traditional materials [6]. These materials not only mimic the natural tooth structure better but also exhibit enhanced durability and resistance to staining, ensuring long-term performance [6]. For prosthodontics, nanotechnology has led to the creation of stronger and more aesthetic dental crowns, bridges and dentures [5]. Nanoparticles can be incorporated into the framework of these prostheses, resulting in a smoother surface, better resistance to bacterial adhesion and more effective fitting, improving both functionality and appearance [6]. Nanotechnology's impact on treatment techniques is profound, particularly in endodontics and periodontics [7]. In endodontic therapy, nanoparticles are utilized in root canal disinfection, ensuring that bacteria are eradicated more effectively than with traditional methods. These nano-antimicrobial agents can penetrate deeply into infected areas, preventing reinfection and promoting faster healing [8]. In periodontal treatment, nanotechnology is used in the development of nanoparticle-based drug delivery systems [9].

These systems can release antimicrobial agents directly to the site of infection, reducing inflammation and accelerating tissue regeneration. Furthermore, nanostructured surfaces on dental implants are becoming increasingly common. These implants are coated with nanoparticles that improve the osseointegration process, allowing for faster and more successful healing of dental implants [10]. One of the key advantages of nanotechnology in dentistry is its potential to improve biocompatibility [11]. Nanomaterials are often more compatible with the natural biological tissues in the oral cavity. Their small size allows them to interact more effectively with biological systems, reducing the risk of adverse immune reactions. This is particularly important in dental implants, where the success of the procedure relies on the material's ability to integrate with the bone [12]. Additionally, nano-materials are being designed to promote tissue regeneration, which is especially valuable in cases of severe tooth decay, gum disease, or post-surgical recovery. Nanoparticles can deliver growth factors or stem cells to enhance tissue healing, potentially reducing the need for extensive surgical procedures and improving overall patient outcomes [13, 14]. The future of nanotechnology in dentistry is vast and holds the promise of even more ground-breaking advancements. Researchers are exploring the development of self-healing dental materials, which could automatically repair minor cracks or wear. Moreover, the incorporation of nanotechnology in orthodontics, such as nano-coated braces that can speed up tooth movement or reduce discomfort, is already on the horizon [15]. As the field evolves, continued research will likely uncover more innovative applications, ensuring that nanotechnology plays a central role in shaping the future of dentistry. From improving dental materials and diagnostics to revolutionizing treatment methodologies and enhancing patient care, nanotechnology is poised to redefine clinical practices, offering more effective, efficient and patient-friendly dental solutions [16]. The applications of nanotechnology in various dentals specialities are given in Table 1.

## Nanotechnology in dental diagnosis:

Nanotechnology has greatly enhanced diagnostic capabilities in dentistry, allowing for earlier, more precise detection of dental diseases [17]. Utilizing the unique properties of nano-scale materials, such as quantum dots, nano-biosensors and nanoparticles, these technologies detect oral pathogens and disease biomarkers even at early stages [18]. By improving the sensitivity and specificity of diagnostic tools, nanotechnology empowers clinicians to identify conditions that may not be visible through conventional methods, enabling earlier intervention and reducing the risk of disease progression [19]. Early detection of oral cancer is challenging due to its occurrence in hidden anatomical areas and the resemblance of precancerous lesions, like oral leukoplakia and erythema, to chronic conditions such as lichen planus. Nanotechnology, particularly gold nanoparticles (AuNPs), has revolutionized diagnostic methods, offering high stability, biocompatibility and enhanced specificity 20. Techniques like Optical Coherence Tomography (OCT) utilize AuNPs for high-resolution imaging of subsurface tissues, while Surface-Enhanced Raman Spectroscopy (SERS) leverages them to amplify molecular signals, distinguishing malignant from normal tissues. Additionally, nano-based MRI contrast agents provide precise imaging by targeting cancer-specific markers, making these advancements vital for early oral cancer diagnosis [20].

Additionally, nanotechnology in imaging, such as through quantum dots or gold nanoparticles, has significantly improved the resolution and clarity of dental scans. These advances enable more precise diagnosis, allowing dental professionals to detect abnormalities in tissues with much greater detail, enhancing treatment planning and outcomes [21]. Quantum dots are semiconductor nanoparticles that exhibit unique optical properties, including fluorescence, which makes them valuable for high-resolution imaging [22, 23]. Due to their small size and high brightness, quantum dots provide excellent contrast in imaging, allowing for detailed visualization of dental tissues. This is particularly beneficial in identifying precancerous lesions, early-stage caries and other pathologies that may not be detected through traditional imaging techniques. Salata (2004) [24] highlights the versatility of nanoparticles like quantum dots in biological imaging and their potential in enhancing medical and dental diagnostics. Nano-biosensors are another breakthrough enabled by nanotechnology, as they allow for real-time detection of oral pathogens. These sensors are typically designed to bind selectively with disease-specific biomarkers, providing rapid and precise readings of bacterial presence. For instance, nanosensors in periodontal diagnostics can detect bacteria associated with gum disease, such as Porphyromonas gingivalis, at very low concentrations. Early detection of such pathogens allows for timely intervention, which can prevent disease progression and reduce the need for invasive treatments later on [23].

## Antimicrobial nanoparticles for diagnostic and therapeutic purposes:

Nanoparticles, particularly silver nanoparticles, have gained attention for their antimicrobial properties. Rai *et al.* (2012) [25] discuss the efficacy of silver nanoparticles against multidrug-resistant bacteria, underscoring their potential to combat oral pathogens resistant to traditional antibiotics. Nanoparticles can be integrated into biosensors or imaging agents that not only detect bacteria but also reduce bacterial load in the diagnostic sample. Silver nanoparticles, in particular, are powerful against a broad spectrum of pathogens and can be used to detect and limit the spread of infection, making them suitable for applications in both diagnostic and preventive dentistry [24, 25]. As nanotechnology progresses, further innovations are anticipated, such as multifunctional nanosensors that combine diagnostic and therapeutic functions, potentially offering real-time monitoring and control of disease progression [25, 26].

## Nanotechnology in restorative dentistry:

Nanotechnology is reshaping restorative dentistry by offering stronger, more durable and aesthetically pleasing materials. The use of nanotechnology in restorative dentistry has ushered in a new wave of advancements, significantly improving the functionality and effectiveness of dental materials. Nano-glass ionomers, combining traditional glass ionomers with nanoparticles offer improved translucency, compressive strength, better elasticity, improved polishability, wear resistance and sustained fluoride release. Similarly, nanocomposites with fillers like silica, zirconia and titanium exhibit superior mechanical strength, reduced polymerization shrinkage and better esthetics, while bioactive and antimicrobial nanoparticles contribute to remineralization and plaque resistance. These advancements collectively enhance the durability and clinical outcomes of restorative treatments [26]. Nanocomposites, which integrate nano-sized fillers, represent a major breakthrough in restorative materials. These nano-fillers improve the physical properties of the composite resin, addressing issues such as wear resistance, polymerization shrinkage and aesthetic qualities. Traditional composites tend to shrink during curing, which can lead to gaps, micro-leakages and ultimately a shorter lifespan for restorations. In contrast, nanocomposites are engineered to reduce this shrinkage, ensuring a more secure fit and minimizing the risk of secondary decay [27]. Nano-sized fillers not only enhance the wear resistance of restorations but also increase the strength and durability of the material, making it less prone to chipping or cracking. This feature is particularly valuable for patients with high occlusal forces, as the materials can better withstand the stresses of chewing and grinding over time [27]. Due to the small size of nanoparticles, these materials can achieve a high level of translucency and polishability, closely matching the natural appearance of teeth [28, 29]. Nanoparticles scatter light in a way that mimics enamel, enabling restorations to blend seamlessly with surrounding tooth structure. This has revolutionized the possibilities for cosmetic restorations, as clinicians can now achieve lifelike results that remain resilient and maintain their aesthetic appeal over time. Nanocomposites represent a durable and versatile option for a wide range of restorative applications, from simple fillings to more complex cosmetic reconstructions. Their enhanced properties improve patient satisfaction, reduce the frequency of repairs or replacements and support long-term oral health. The potential for further advancements in nanotechnology-based restorative materials continues to grow, with on-going research focused on developing self-healing composites and multifunctional materials that can respond to oral pH changes or release therapeutic agents [30, 31].

## Nanotechnology in endodontics:

Nanotechnology plays a transformative role in endodontics by enhancing root canal disinfection through the use of nanoparticles with potent antimicrobial properties. Traditional root canal treatments often face challenges in fully eradicating bacteria, particularly in complex canal structures with narrow and intricate branches. Nanoparticles, including silver and zinc oxide, have shown exceptional efficacy in eliminating bacteria that may be resistant to standard disinfectants, offering a more reliable solution to reduce the risk of reinfection [32]. Silver nanoparticles are highly valued in endodontics for their potent antibacterial effects. When incorporated into irrigants or sealers, silver nanoparticles provide sustained antimicrobial action, penetrating deep into dentinal tubules and reaching areas that conventional disinfectants may miss. Due to their small size and large surface area, silver nanoparticles can interact more effectively with bacterial cell walls, disrupting bacterial function and ultimately leading to cell death. This makes them particularly useful for targeting bacteria embedded in biofilms, which are notoriously resistant to standard root canal disinfectants [33].

Zinc oxide nanoparticles are another valuable addition to root canal disinfection. Known for their broad-spectrum antimicrobial activity, these nanoparticles are effective against a wide range of oral pathogens, including *E. faecalis*, a common bacteria found in failed root canal treatments. When used in endodontic sealers or irrigants, zinc oxide nanoparticles help to ensure that even difficult-to-reach areas within the canal are thoroughly disinfected [34]. Their long-lasting antibacterial action provides additional protection post-treatment, reducing the risk of residual infection and contributing to better long-term outcomes for endodontic procedures. Another key benefit of using nanoparticles for root canal disinfection is their ability to penetrate the complex microstructures within the canal system. This includes lateral canals, isthmuses and dentinal tubules, where bacteria often hide and persist. Nanoparticles can navigate these structures more effectively than traditional irrigants, reaching areas that are difficult to access [35]. Furthermore, studies have shown that these nanoparticles are generally biocompatible, meaning they can be safely used in endodontic treatments without harming surrounding tissues. As research advances, the development of multifunctional nanoparticles that combine antimicrobial and regenerative properties is a promising area. Such nanoparticles could aid in disinfection while simultaneously promoting tissue healing and regeneration. This approach may reduce treatment time and improve success rates by addressing both microbial eradication and tissue recovery in a single step [36, 37].

## Nanotechnology in removable prosthetics:

Nanotechnology is transforming prosthodontics by addressing the limitations of traditional materials like Polymethyl methacrylate. While Polymethyl methacrylate is widely used for denture bases due to its affordability, biocompatibility and aesthetics, its mechanical weaknesses can be mitigated through reinforcement with nanoscale agents such as metal oxides, noble metals and carbon nanoparticles. Studies have demonstrated that incorporating nanoparticles like TiO2 and zirconium oxide into PMMA improves compressive strength, antimicrobial properties and longevity, while enhancing biocompatibility and structural features [38]. Innovations in nanoparticle coatings for dentures, soft liners and cement further improve antimicrobial activity, bond strength and mechanical performance, offering promising advancements in durability, oral health outcomes and cost-effectiveness.

## Nanotechnology in implantology:

Nanotechnology has led to significant innovations in dental implants, particularly through the development of nano-modified surfaces that enhance osseointegration and reduce bacterial colonization. Nanoscale modifications on implant surfaces improve cellular responses, promoting better cell adhesion and stability, which are essential for successful bone integration and long-term implant success [39]. These advancements in implant surface technology provide a dual benefit of strengthening the bone-implant connection while reducing the likelihood of peri-implant infections. Traditional implant surfaces are generally roughened to improve bone contact, but nano-modified surfaces incorporate nanoscale topographies. These nanoscale modifications mimic natural bone structure, promoting the adhesion and proliferation of osteoblasts (bone-forming cells). This enhances the bone-implant contact area, speeding up the osseointegration process and improving the initial stability of the implant, which is crucial for long-term success and patient comfort [40].

Titanium implants with nano-modified surfaces have been shown to significantly improve bone-implant contact and enhance mechanical stability. Studies indicate that these nano-surfaces encourage osteoblast attachment and increase the rate of new bone formation around the implant, leading to faster and more secure integration with the surrounding bone. Another key benefit of nano-engineered implant surfaces is their ability to minimize bacterial adhesion, which is essential in preventing peri-implantitis, a common cause of implant failure [41]. Bacteria can quickly colonize the surface of implants after placement, leading to inflammation and infection around the implant site. Nano-modified surfaces are designed to reduce bacterial attachment while supporting beneficial cellular responses. This antimicrobial property decreases the risk of peri-implantitis, improving the implant's long-term prognosis [42]. Implants coated with silver or other antimicrobial nanoparticles show enhanced resistance to bacterial colonization without compromising osseointegration. These surfaces create an environment that supports tissue growth while deterring the adhesion of harmful microbes, offering a proactive approach to infection control at the implant site. Nanotechnology in implantology is an evolving field, with on-going research focused on multifunctional surfaces that combine osteogenic (bone-forming) and antimicrobial properties. Future advancements may include smart implant surfaces that respond to changes in the surrounding tissue environment, releasing antimicrobial agents which promote bone growth. These innovations hold the potential to further improve patient outcomes, reduce complications and extend the functional lifespan of dental implants [43].

## Nanotechnology in periodontics:

In periodontics, nanotechnology has introduced highly effective methods for targeted drug delivery, improving the treatment of periodontal diseases by enabling precise and controlled release of therapeutic agents directly to affected periodontal tissues. Traditional treatments often involve systemic administration of anti-inflammatory or antimicrobial agents, which can lead to widespread effects throughout the body and limited impact on localized infection sites. Nanoparticles address this by providing a targeted approach that minimizes systemic exposure, focusing therapeutic effects where they are most needed and thereby enhancing treatment outcomes [44]. One of the key applications of nanotechnology in periodontics is the use of polymer-based nanoparticles for localized drug delivery within periodontal pockets. These nanoparticles can be loaded with anti-inflammatory or antimicrobial agents and designed to release their contents slowly over time. This controlled release allows for a steady concentration of therapeutic agents directly at the infection site, maximizing effectiveness while reducing the frequency of applications and potential side effects associated with high-dose systemic treatments [45].

For example, polymer-based nanoparticles carrying antimicrobials or anti-inflammatories can be applied directly to periodontal pockets affected by periodontitis. The nanoparticles provide a sustained release of the agents, targeting bacteria and inflammation at the infection site. This approach not only enhances the eradication of pathogens but also supports healing by maintaining a stable environment within the periodontal pocket. The localized and sustained release of drugs from nanoparticles is especially beneficial in managing periodontitis, a chronic inflammatory condition driven by bacterial infection of periodontal tissues. By focusing on the affected sites, nanoparticle-based treatments reduce the need for high systemic doses, lowering the risks of side effects and resistance associated with repeated antibiotic use. Additionally, the precise delivery helps maintain a longer therapeutic window within the periodontal pocket, improving the control of both inflammation and bacterial load [46].

Nanoparticles can be tailored to carry multiple agents, allowing for combination therapies within a single application. They may deliver both an antimicrobial to control infection and an anti-inflammatory to reduce tissue damage, addressing the multiple facets of periodontitis simultaneously. This approach supports better tissue healing and regeneration, providing patients with improved results and a more effective management plan for periodontal disease. Looking ahead, nanotechnology may further advance periodontal treatments by integrating regenerative therapies. Researchers are developing nanoparticles that carry growth factors or stem cell stimulators, which may promote tissue regeneration in addition to combating infection. These multifunctional nanoparticles could play a transformative role in reconstructing lost periodontal tissue and restoring the structural integrity of the periodontal complex, advancing from simply managing symptoms to reversing damage caused by periodontitis.

## Nanotechnology in orthodontics:

Nanotechnology has revolutionized orthodontics by enhancing materials and techniques for improved treatment outcomes. Nano-coatings like ZnO reduce archwire friction by up to 51%, improving movement efficiency, while nano-filled composites enhance shear bond strength and antimicrobial properties using materials like silver and hydroxyapatite [47]. Additionally, nanoparticles in adhesives and coatings, including ZnO and Ag, prevent bacterial growth, ensuring better durability and patient comfort. These advancements reduce treatment duration and improve precision, marking a significant leap in orthodontic care.

## Nanotechnology in tissue engineering:

Nanotechnology has become a powerful tool in tissue engineering, significantly advancing the design and functionality of scaffolds used for dental and bone tissue regeneration. Nano-engineered scaffolds are meticulously crafted to mimic the natural extracellular matrix (ECM), providing an environment conducive to cell growth, adhesion and differentiation. These properties are essential for successful tissue regeneration, particularly in complex structures such as bone and dental tissues. By simulating natural tissues, nanotechnology-based scaffolds not only promote cellular activities but also enhance the integration and stability of newly formed tissues, paving the way for more effective regenerative treatments in dentistry and maxillofacial surgery [48]. Hydroxyapatite nanoparticle scaffolds can be used in bone grafting procedures during maxillofacial surgery, offering a framework that not only fills defects but actively promotes the regeneration of native bone [49]. These scaffolds have high biocompatibility and bioactivity, integrating seamlessly with existing bone tissue and supporting cellular activities that are critical for bone healing.

Nano-engineered scaffolds are also applied to regenerate tissues such as periodontal ligament and dentin. The scaffolds are often made from biomimetic materials, structured at the nanoscale to mimic the natural architecture of dental tissues. These nano-scaffolds provide a supportive environment for dental pulp cells, stem cells and other regenerative cells, aiding in the formation of dentin and other supportive structures necessary for tooth health [50]. Nano-scaffolds can be loaded with growth factors or signalling molecules that encourage stem cells to differentiate into specific cell types, such as odontoblasts for dentin formation. The precision and control afforded by nanotechnology enable the development of scaffolds that promote tissue-specific regeneration, creating opportunities for improved outcomes in restorative dentistry, especially for cases involving significant tissue loss. Beyond structural support, nano-engineered scaffolds can be further enhanced to release bioactive agents, such as antimicrobial or anti-inflammatory drugs, as well as growth factors that support tissue regeneration. These multifunctional scaffolds can provide sustained therapeutic benefits, actively promoting regeneration while protecting against infection, a common concern in oral and maxillofacial surgery. Research is on-going into developing "smart" scaffolds that respond to the tissue environment, adjusting their properties as regeneration progresses to optimize healing.

## Conclusion:

The application of nanotechnology in dentistry enhance diagnostic precision, improve materials in restorative dentistry, support effective endodontic disinfection, promote implant osseointegration and enable localized drug delivery in periodontics and advance tissue engineering strategies. Continued research and innovation in this field promise to shape the future of dentistry, making treatments more efficient, less invasive and highly patient-friendly.

## Figures and Tables

**Table 1:** Summarizing the applications of nanotechnology across various dental specialties

**Dental Specialty**	**Nanotechnology Application**	**Example**	**Key Benefits**
Dental Diagnosis	Quantum dots, nano-biosensors and nanoparticles for early detection of oral pathogens and disease biomarkers.	Nanosensors for detecting bacteria in periodontal disease.	Early and accurate diagnosis, allowing for timely intervention and improved patient outcomes.
Restorative Dentistry	Development of smart composites with nano-sized fillers that enhance durability, wear resistance and aesthetics.	Nano-filled smart composites reduce polymerization shrinkage and improve wear resistance.	Enhanced longevity and aesthetics in restorations.
Endodontics	Nanoparticles with antimicrobial properties for effective root canal disinfection, especially in complex canal structures.	Silver nanoparticles in irrigants or sealers provide superior antibacterial effects.	Improved root canal disinfection, reducing the risk of reinfection.
Implantology	Nano-modified implant surfaces to enhance osseointegration and reduce bacterial colonization.	Titanium implants with nano-textured surfaces improve bone-implant contact and reduce peri-implantitis risk.	Improved stability, integration with bone and lower infection rates.
Periodontics	Targeted drug delivery via nanoparticles to control release of anti-inflammatory or antimicrobial agents within periodontal pockets.	Polymer-based nanoparticles for localized drug release in periodontitis treatment.	Minimizes systemic effects, enhances localized treatment effectiveness and reduces frequency of applications.
Tissue Engineering	Nano-engineered scaffolds that mimic natural tissues, supporting cell growth and differentiation for dental and bone tissue regeneration.	Hydroxyapatite nanoparticles create a matrix for bone regeneration in maxillofacial surgery.	Promotes regeneration, enhances cell adhesion and improves integration of newly formed tissues.
